# Supplementary material for: Modelling indirect interactions during failure spreading in a project activity network
Source: Sci Rep. 2018 Mar 12;8:4373. doi: 10.1038/s41598-018-22770-3 (PMC5847592; doi:10.1038/s41598-018-22770-3)
Supplement: Supplementary file 1 — Supplementary Material [file 41598_2018_22770_MOESM1_ESM.pdf]

Supplementary Material for ‘Modelling indirect interactions during failure spreading in a project activity network’

Christos Ellinas[1,*]

[1] *Engineering Mathematics, University of Bristol, Bristol, UK*

[*]Corresponding author

E-mail: ce12183@bristol.ac.uk

1. Visualisation of empirical activity network

Supplementary Fig. 1 visualises the empirical activity network, with subplot (b) focusing on an exemplar spreading event which includes several instances of non-tree-like behaviour – this behaviour is highlighted by grey nodes. Notice the resemblance of the highlighted topology with the motivational example used in Fig.1 of the main paper.

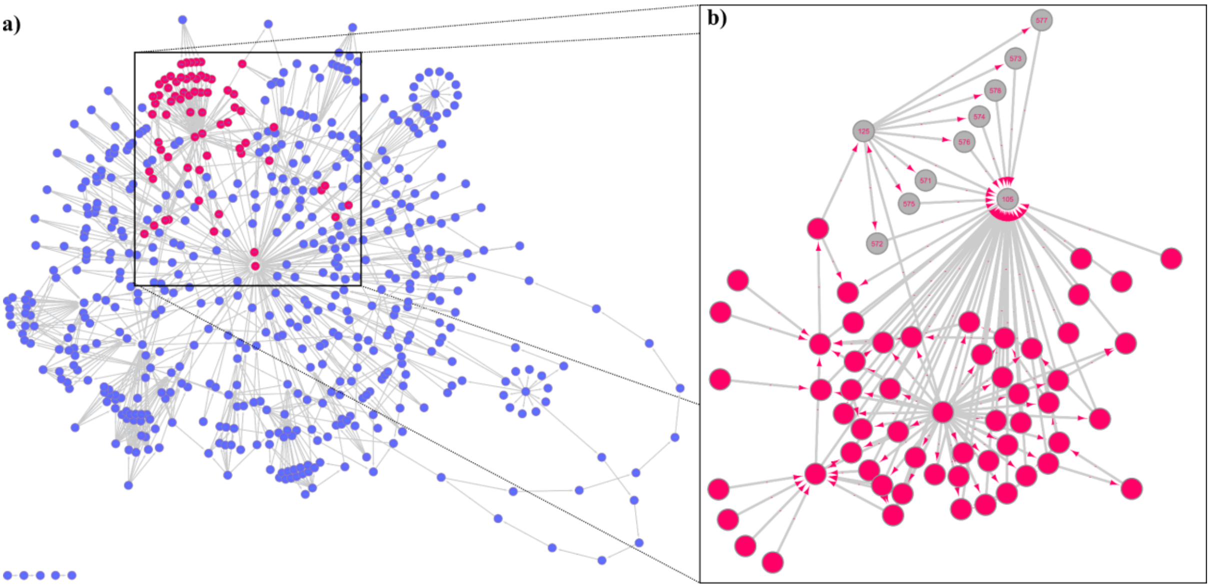

Supplementary Fig. 1: Visualisation of (a) the empirical activity network, highlighting an exemplar spreading event (red nodes), (b) exemplar spreading event, highlighting the deviation from tree-like behaviour (grey nodes). Note the resemblance to the example used in Fig. 1.

2. Tree network case

The average spreading event size obtained under model M_0 (Supplementary Fig. 2a) is visually indistinguishable from those obtained under model M (Supplementary Fig. 2b). This is increasingly evident once we consider r^{avg} (Supplementary Fig. 2c), the results of which converge to a value of 1, with some normally distributed noise that results from the stochastic nature of the simulation (Supplementary Fig. 2d). This coherence between model M_0 and M illustrates the lack of an effect from indirect exposures, which is to be expected, since non-trivial subgraphs such as the ones noted in Figure 1, as they are explicitly forbidden by the very structure of the tree network.

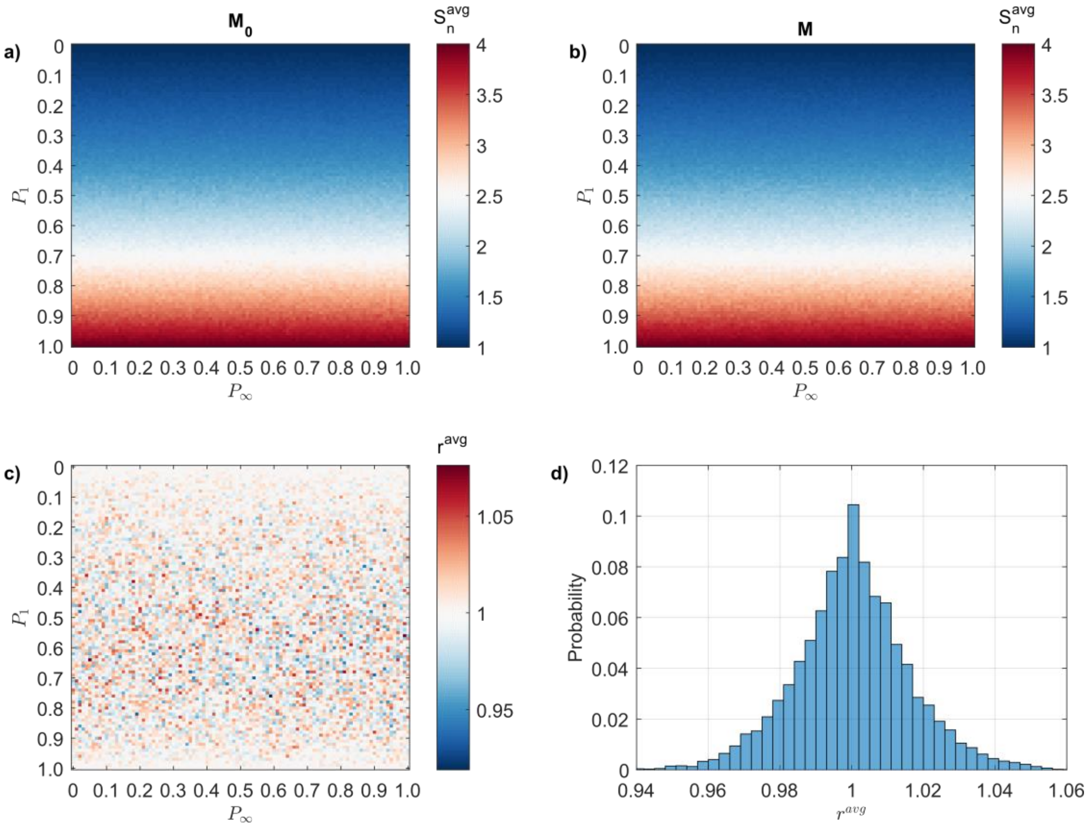

Supplementary Fig. 2: (a) spreading event size across the parameter space under model M_0; (b) same as (a) under model M; (c) value r^{avg} across the parameter space, demonstrating the lack of an effect as indirect exposure increases; (d) histogram of r^{avg} values in (c),converging to a value of 1 (no difference between M_0 and M) with some normally distributed noise due to the stochastic nature of the simulation.

3. Example subgraphs for varying S_d and S_w

Supplementary Fig. 3 provides simple examples for varying S_d and S_w used to characterise the topology of the spreading pathways, whilst preserving the size of the spreading event (S_n) constant (in this case, $S_n = 5$). Nodes that contribute to the final S_d and S_w are marked using dotted and blue borders, respectively

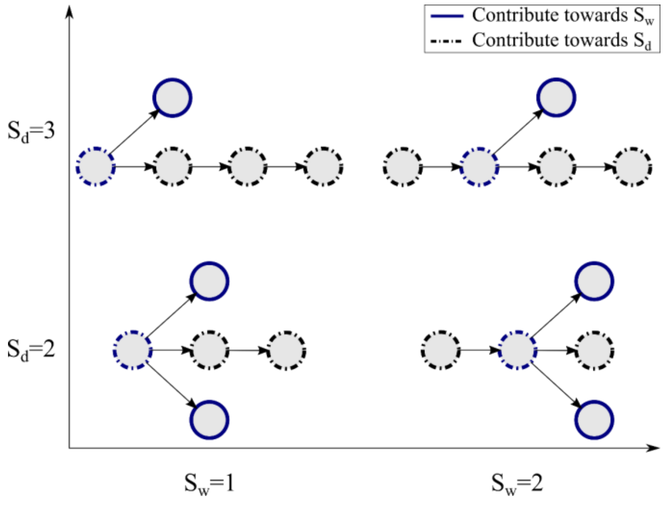

Supplementary Fig. 3: A variety of spreading pathways that capture the failure of six nodes, as characterised by varying S_d and S_w.

4. Average Spreading Event Size

Similar to the case of $S_n^{\max}$ reported in the main manuscript (Figure 6), a similar relationship between $S_n^{\max}$ and $S_{d/w}$ is noted, where small to medium events rely on narrow pathways (high value of $S_{d/w}$), see Supplementary Fig. 4a and 4b respectively. Larger events are subsequently enabled by branching out to include additional pathways which result to wider pathways (low value of $S_{d/w}$).

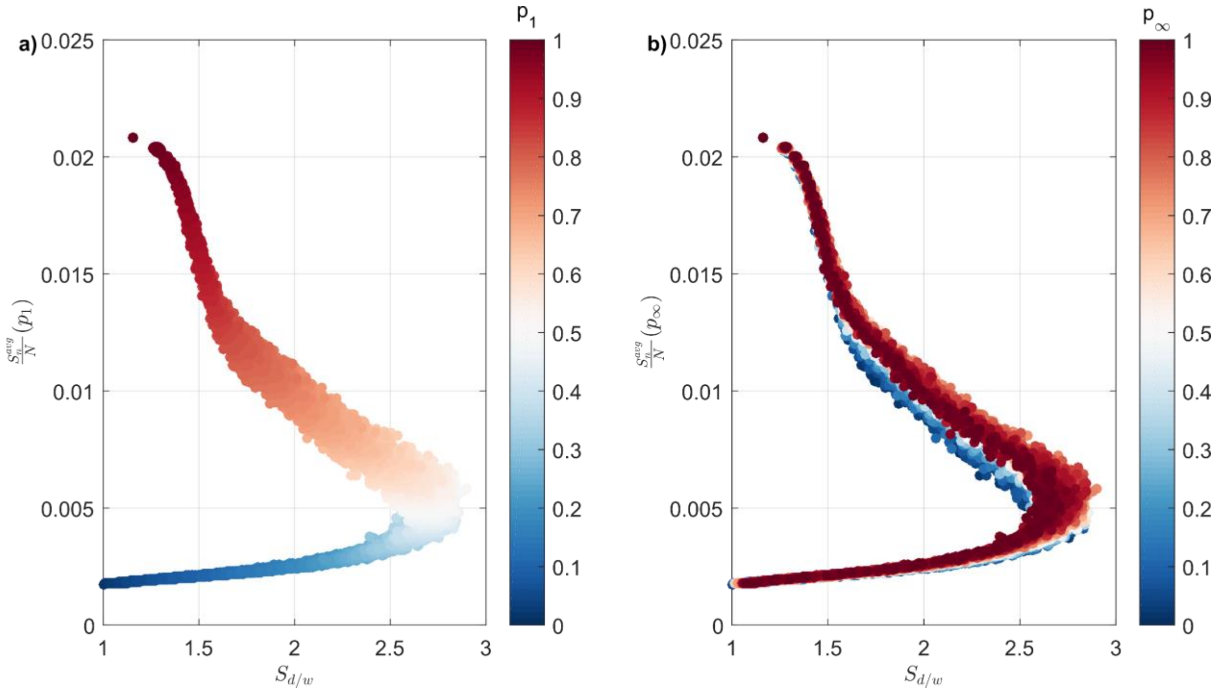

Supplementary Fig. 4: Parameter space for the average spreading event size $\left(S_n^{\text{avg}}\right)$, normalised over the total number of nodes (N), and its underlying structure $\left(S_{d/w}\right)$, as a function of (a) p_1 and (b) p_∞.

5. Influence of p_1 and p_∞ to average propagation rate

Focusing on the relationship between the propagation rate of the largest spreading event, $S_r^{\max}$, and the structure of the pathways used to sustain it, $S_{d/w}$, Supplementary Fig. 5 and Supplementary Fig. 6 map the results obtained under discrete intervals of 0.2 for p_1 and p_∞ respectively. Focusing on the effect of indirect exposure, the fork-like behaviour – and the associated presence of two possible trajectories – exist under the limited range of p_∞ values, roughly ranging from 0 to 0.6 (Supplementary Fig. 6). Beyond these values, the two possible trajectories reduce to one, demonstrating the principal role of indirect exposure in controlling the emergence of the two pathways. For comparison, direct exposure has limited control over the emergence of this fork-like behaviour, with p_1 being unable to trigger the definitive emergence of the two trajectories (Supplementary Fig. 5).

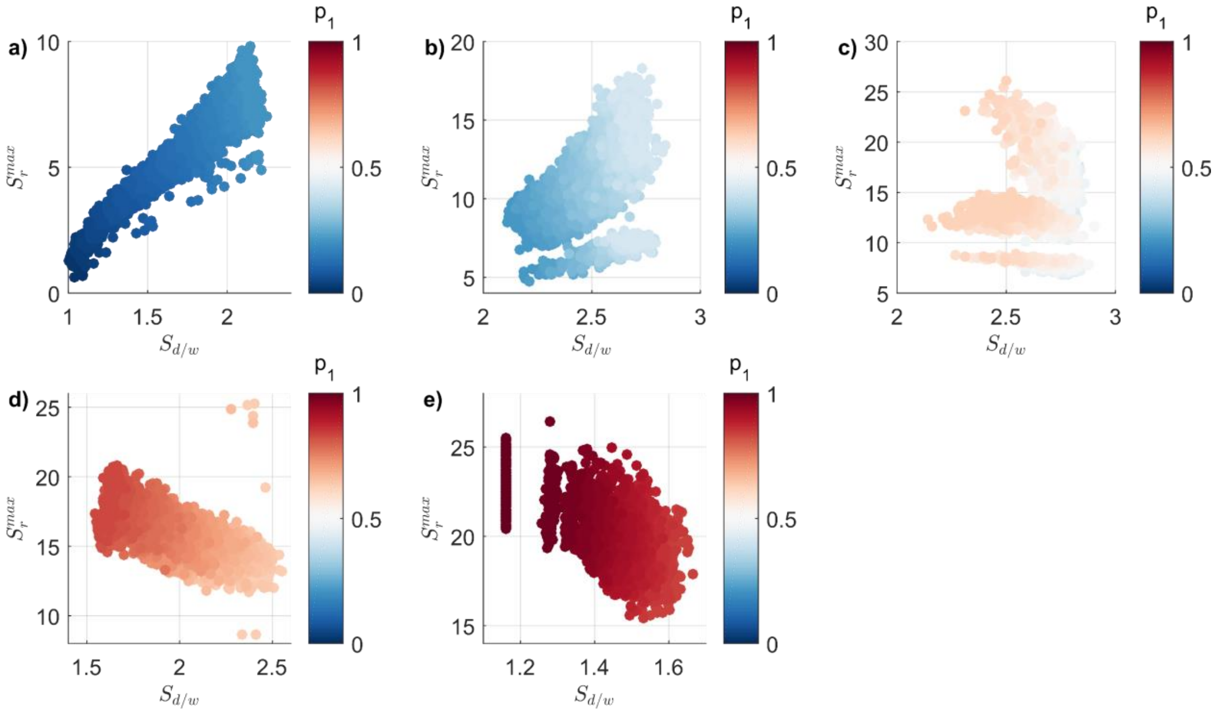

Supplementary Fig. 5: Parameter space for the relationship between the propagation rate of the largest spreading event, $S_r^{\max}$, and the structure of the pathway sued to sustain it, $S_{d/w}$, as a function of p_1, at 0.2 intervals, ranging from $p_1 \in [0,0.2]$ in subplot (a) – to $p_1 \in [0.8,1]$ in subplot (e).

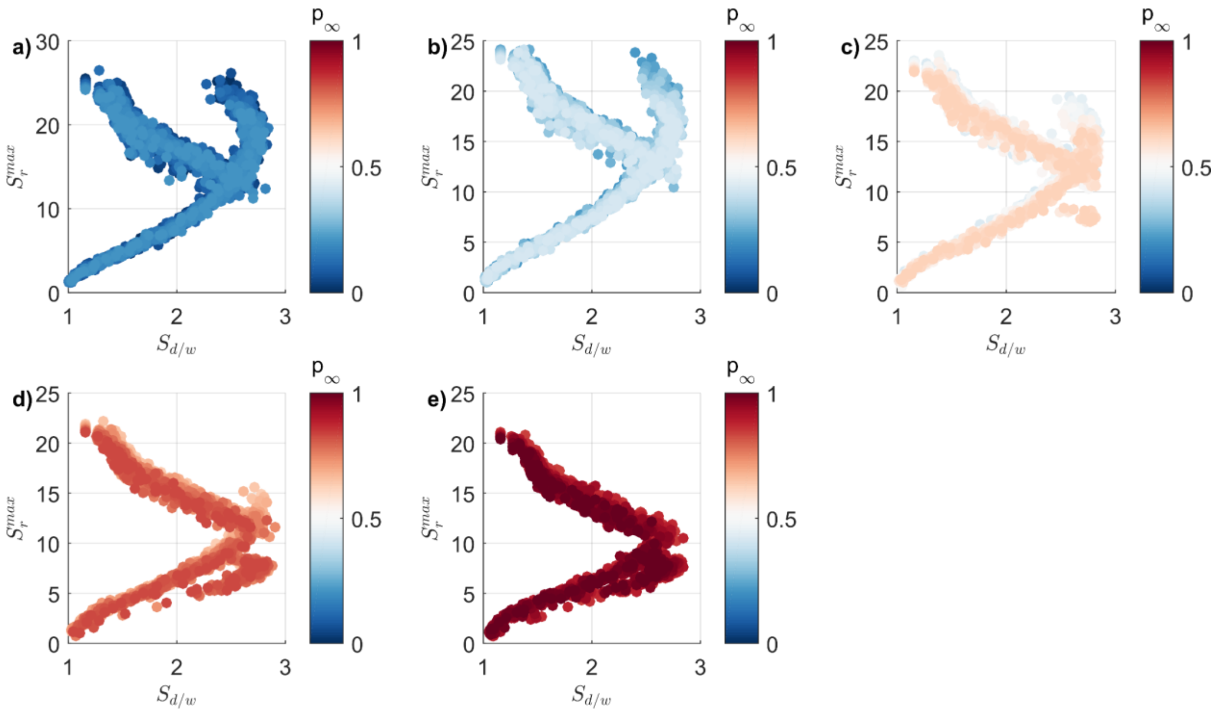

Supplementary Fig. 6: Parameter space for the relationship between the propagation rate of the largest spreading event, $S_r^{\max}$, and the structure of the pathway sued to sustain it, $S_{d/w}$, as a function of p_∞, at 0.2 intervals, ranging from $p_\infty \in [0,0.2]$ in subplot (a) – to $p_\infty \in [0.8,1]$ in subplot (e).

6. Degree distribution of empirical network

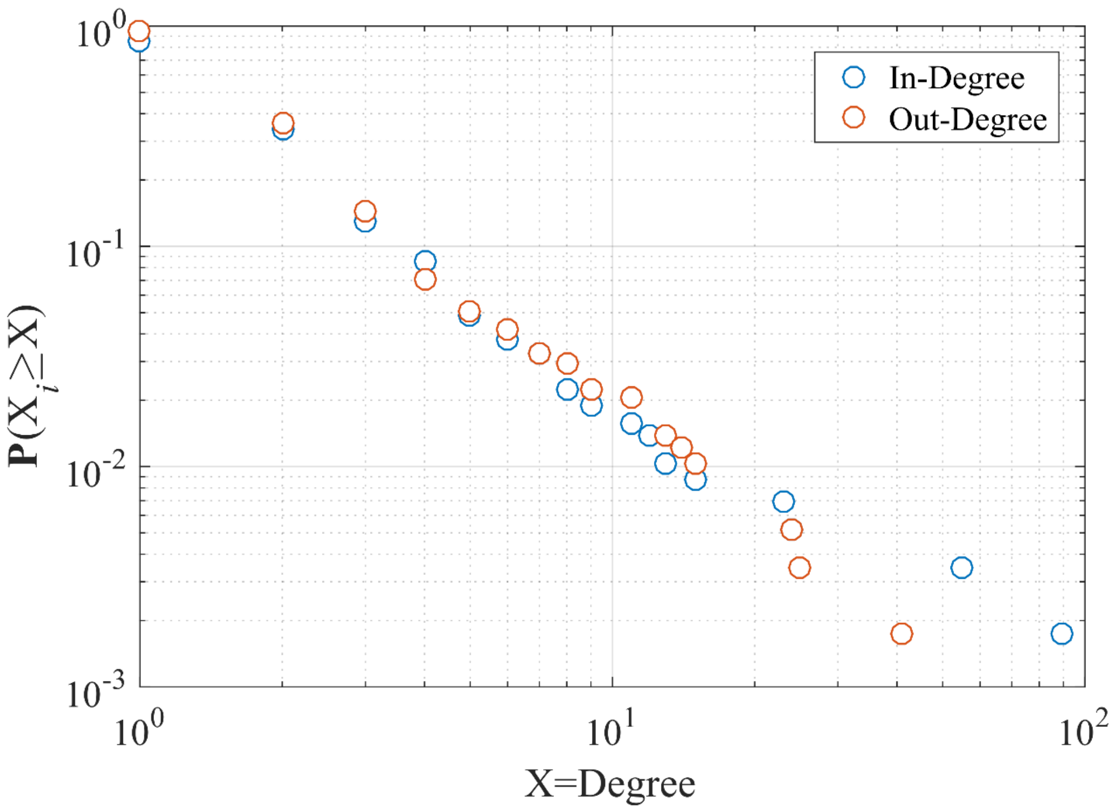

Supplementary Fig. 7: Cumulative probability distribution for in-degree (blue) and out-degree (red) of the activity network. Note the heavy-tail nature of both distributions, evident by the straight plot line under log-log axes.
